# The attentional boost effect and perceptual degradation: Assessing the influence of attention on recognition memory

**DOI:** 10.3389/fpsyg.2022.1024498

**Published:** 2022-11-18

**Authors:** Mitchell R. P. LaPointe, Tamara M. Rosner, Javier Ortiz-Tudela, Lisa Lorentz, Bruce Milliken

**Affiliations:** ^1^Department of Psychology, Neuroscience & Behaviour, McMaster University, Hamilton, ON, Canada; ^2^Department of Psychology, Mount Allison University, Sackville, NB, Canada; ^3^Facultad de Psicologia, Universidad de Granada, Granada, Spain; ^4^Institüt fur Psychologie, Goethe Universität, Frankfurt, Germany

**Keywords:** attention, recognition memory, attentional boost effect, divided attention, degradation effect

## Abstract

Researchers have suggested that the recognition memory effects resulting from two separate attentional manipulations—attentional boost and perceptual degradation—may share a common cause; namely a transient up-regulation of attention at the time of encoding that leads to enhanced memory performance at the time of retrieval. Prior research has demonstrated that inducing two similar transient shifts of attention simultaneously produces redundant performance in memory. In the present study, we sought to evaluate the combined influence of the attentional boost and perceptual degradation on recognition memory. If these two effects share a common cause, then we ought to observe a redundancy in memory performance, such that these two factors interact. Yet, across four experiments we fail to observe such a redundancy in recognition memory. We evaluate these results using the limited resource model of attention and speculate on how combining transient shifts of attention may produce redundant memory performance in the one case, but non-redundant performance in the other case.

## Introduction

It is axiomatic that attention plays an important role in remembering—“paying attention” improves remembering, and divided attention undermines remembering (e.g., Jacoby et al., 1993; Craik et al., 1996). At the same time, attention is a multifaceted construct (e.g., Posner and Peterson, 1990), and the study of how particular attention processes influence memory is an emerging field of interest. In the present study, we examined the effect of transient shifts of attention on memory encoding, with a particular focus on two distinct effects thought to be produced by such transient attention shifts: the attentional boost effect and the perceptual degradation effect.

### The attentional boost effect

It is well established that engaging in two tasks simultaneously is associated with performance costs (Welford, 1952). These dual task costs are sometimes attributed to attentional resources being limited; use of attentional resources on one task reduces resource availability for a second task (Wickens, 1980). Alternatively, dual task costs have been attributed to a bottleneck occurring at the response selection stage (Pashler, 1994); while one task occupies that processing stage, access for a second task is postponed and thus produces performance costs. Both of these theoretical views have been applied to a wide range of dual task interference effects.

Although dual task performance costs may be the norm, Swallow and Jiang (2010) reported a dual task benefit, where attending to two tasks simultaneously during an encoding phase produced superior memory in a following test phase. In the divided attention trials of their study, participants studied a series of natural images while also performing a secondary detection task. Each natural image was overlaid with a small square for 100 ms—a white square on 20% of trials and a black square on 80% of trials. While studying the images, the secondary task involved detecting white target squares with a button press while ignoring black distractor squares. Importantly, recognition memory of natural images was better on target trials than distractor trials. In fact, recognition on target trials did not differ from a full attention condition in which participants’ only task was to remember the natural images. Swallow and Jiang labelled the surprisingly good memory on divided attention target trials the *attentional boost effect* (ABE). They proposed that detecting a target produces a transient up-regulation of attention during an early phase of encoding that enhances memory for items in close spatiotemporal proximity to the target.

Mulligan et al. (2014) subsequently extended the attentional boost procedure to lexical materials. Word frequency effects in recognition are well established, with the usual finding that recognition is better for low than high frequency words (Gorman, 1961). Mulligan et al. noted that low frequency words may attract more attention than high frequency words in an early phase of encoding (Glanzer and Adams, 1990; Criss and Malmberg, 2008). If this is the case, then word frequency effects in recognition could be driven by the same transient up-regulation of attention that produces the ABE. Mulligan et al. reported a pattern of data consistent with this idea—a robust ABE for high frequency but not for low frequency words.

### The perceptual degradation effect

Several studies have demonstrated that increased perceptual processing difficulty can result in improved memory (Nairne, 1988; Hirshman and Mulligan, 1991; Hirshman et al., 1994; Mulligan, 1996; Mulligan, 1999; Diemand-Yauman et al., 2011; Rosner et al., 2015; although see Yue et al., 2013), in line with the desirable difficulty principle (Bjork, 1994). One account of these processing difficulty effects is that, like low frequency words and target detection, processing difficulty results in a transient up-regulation of attention that improves memory encoding. For example, Rosner et al. had participants read an intermixed list of clear and blurry words in an incidental study phase. Participants then completed a recognition test for the words they had previously read. Recognition sensitivity was better for blurry than clear words. Rosner et al. suggested that a transient up-regulation of attention may have strengthened memory encoding and consequently improved recognition for blurry items.

The purpose of the present study was to evaluate the relation between perceptual degradation and attentional boost effects on recognition by examining them together. We were particularly interested in whether transient shifts of attention on ‘boost’ (target present) trials and on blurry trials would be redundant, as appears to be the case for ‘boost’ and low frequency trials (Mulligan et al., 2014). If target detection and perceptual degradation produce redundant transient shifts of attention, then we should observe a larger ABE for clear than for blurry words.

## Experiments 1a and 1b

To measure both the attentional boost and perceptual degradation effects in Experiment 1a, participants read clear and blurry words while monitoring for and responding to target signals. According to Swallow and Jiang (2010), the ABE reflects an up-regulation of attention to items in close spatiotemporal proximity to targets that strengthens memory encoding. Rosner et al. (2015) proposed a similar account for the perceptual degradation effect—a transient up-regulation of attention for blurry items strengthens memory encoding. Our goal was to examine whether these two attention manipulations produce redundant effects on recognition. If so, then the attentional boost effect should be larger for clear than blurry words.

Experiment 1b was conducted as a full attention control condition. Participants read clear and blurry words, but were told nothing about the target and distractor boost signals. In this experiment, we should observe a perceptual degradation effect, but no ABE. Furthermore, performance for the target present trials in Experiment 1a should approximate that of the corresponding full attention condition in Experiment 1b (e.g., Swallow and Jiang, 2010; Mulligan et al., 2014).

### Method

#### Participants

For all experiments in this article, participants were recruited from a pool of undergraduate psychology students at McMaster University. Participants gave informed consent in accordance with the McMaster Research Ethics Board, reported normal or corrected-to-normal vision, and were compensated $10 CAD or partial course credit. A power analysis conducted using G*Power 3 (Faul et al., 2007) aimed at measuring a large effect size (Cohen, 1988; Mulligan et al., 2014) with power = 0.80 revealed that 20 participants were required. For counterbalancing purposes, we collected data from 24 participants in both experiments. An *a posteriori* sensitivity analysis for a 2 × 2 repeated measures ANOVA assuming power = 0.80 revealed that we could reliably measure effect sizes larger than *f* = 0.41. Twenty-four undergraduates (20 females) ranging in age from 18 to 23 years (*M* = 19.17, *SD* = 1.24) participated in Experiment 1a. A separate group of 24 undergraduates (17 females) ranging in age from 18 to 22 years (*M* = 18.92, *SD* = 1.10) participated in Experiment 1b.

#### Apparatus and stimuli

**Figure 1:**
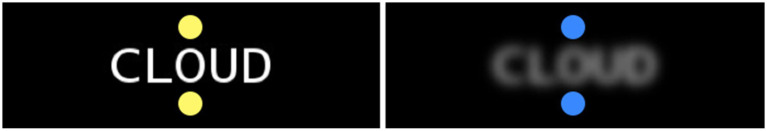
An example of a clear and blurry word, including target and distractor coloured dots, used in each of the four experiments.

Stimuli were presented on a 24-inch BENQ LCD monitor with a resolution of 1920 × 1,080 pixels, using PsychoPy software (Peirce, 2007, 2009). Manual responses were recorded using a QWERTY keyboard. The stimuli consisted of five letter high-frequency nouns (Kučera and Francis, 1967). The words subtended 4.01° of visual angle horizontally and 0.92° vertically. Clear words were presented as text stimuli using PsychoPy. Blurry words were created by applying a Gaussian blur radius of 15 pixels to each word using GNU Image Manipulation Program (GIMP).^1^ Rosner et al. (2015) demonstrated that this level of degradation reliably produces perceptual disfluency effects in recognition of single words [see also Xie et al. (2018); Weissgerber et al. (2021) for an interesting recent debate about perceptual disfluency effects in recall of text]. The blurry words were imported into PsychoPy as picture files. On each trial of the study phase, one coloured dot appeared above the word and a second coloured dot appeared below the word. Each dot had a diameter that subtended 1.15° of visual angle, and both dots were either blue or yellow on any given trial. Examples of these stimuli are presented in Figure 1.

#### Procedure

Participants were seated approximately 50 cm from the computer monitor. Each experiment consisted of a study phase, a distractor phase, and an incidental memory test phase. For Experiments 1a and 1b, the distractor and test phases were identical; the study phases differed slightly, as described below.

In Experiment 1a, the study phase included 10 practice trials and 120 experimental trials. Each trial began with a fixation cross for 200 ms, followed by a word and two coloured dots. The word was either clear or blurry, and both dots were either blue or yellow. The dots were presented for 100 ms, whereas the word remained on screen for 700 ms. Participants were instructed to: (1) read aloud the word; and (2) monitor the colour of the dots—if the dots matched the target colour, they were to press the spacebar.

In Experiment 1b, the study phase was identical to Experiment 1a with the exception that participants were instructed only to read aloud the word on each trial; they were told nothing about the dots that appeared with the words.

Following the study phase, there was a 10-min distractor task that required completion of arithmetic problems. Finally, the test phase involved an incidental recognition test. Each recognition trial began with presentation of a word. Participants were instructed to press the ‘A’ key for an ‘old’ response and the ‘L’ key, for a ‘new’ response. To assess recollection and familiarity (Rajaram, 1993; Yonelinas and Jacoby, 1995; Yonelinas, 2002) participants also made remember/know judgments following all ‘old’ responses. If participants ‘remembered’ seeing the word in the study phase they were to press the ‘z’ key, whereas if they had a feeling of ‘knowing’ the word had been presented in the study phase they pressed the ‘/’key (McCabe and Geraci, 2009). The remember/know procedure was included for exploratory purposes, and recollection and familiarity estimates and analyses are presented in Appendix A, but not in the body of the paper.

#### Design

The words were drawn from four lists of 60 words (see Appendix B). Of those 240 words, 120 were presented in both the study and test phases (‘old’ words). The remaining 120 words appeared only in the test phase (‘new’ words). For both ‘old’ and ‘new’ words, half appeared clear and half appeared blurry. This constraint was achieved by assigning one of the four lists to each of the clear ‘old’, blurry ‘old’, clear ‘new’, and blurry ‘new’ conditions. The assignment of lists to conditions was counterbalanced across participants such that each list appeared as old/new and clear/blurry an equal number of times. Presentation of words in both the study phase and test phase was randomized. During the test phase, ‘old’ words appeared as they had appeared in the study phase (i.e., if a word was blurry during the study phase, it was also blurry in the test phase). In both Experiments 1a and 1b, targets occurred on a random 20% of trials and distractors occurred on the other 80% of trials. The assignment of colours (blue/yellow) to target and distractor roles was counterbalanced across participants.

### Results

#### Study phase

For Experiment 1a, target detection sensitivity in the study phase was calculated by subtracting the false alarm rate from the hit rate, separately for clear and blurry words. There was no difference in sensitivity to boost targets for clear (*M* = 0.97) and blurry (*M* = 0.94) words (*p* > 0.05). For Experiment 1b, participants did not respond to target signals, so no such comparison was conducted.

#### Test phase

**Figure 2:**
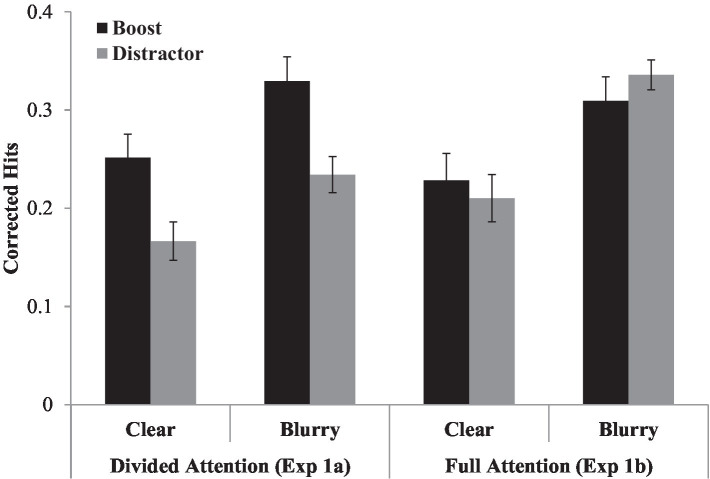
Mean corrected hits (hits minus false alarms) for each condition for both Experiment 1a, in which the task involved word reading and signal monitoring, and Experiment 1b, in which the task involved only word reading. Error bars reflect standard errors corrected to remove overall between-subject variation (Morey, 2008).

The proportion of ‘old’ responses for old and new items (i.e., hits and false alarms) for each condition are presented in Table 1. Corrected hit rates (i.e., hits minus false alarms) were computed for each condition and submitted to separate repeated measures analyses of variance (ANOVA) for each experiment. The ANOVAs treated perceptual degradation (clear/blurry) and boost signal (target/distractor) as within-participant factors. Mean corrected hits for each condition are plotted in Figure 2.

**Table 1:** Proportions of hits and false alarms for each condition in Experiments 1a, 1b, 2a, and 2b. Standard errors corrected by removing overall between-participant variance are presented in parentheses (Morey, 2008).

Exp.	Clear Words			Blurry Words		
	Target	Distractor	FAs	Target	Distractor	FA’s
1a	0.52 (0.02)	0.43 (0.02)	0.27 (0.02)	0.61 (0.03)	0.52 (0.02)	0.28 (0.02)
1b	0.50 (0.03)	0.48 (0.02)	0.27 (0.02)	0.59 (0.03)	0.61 (0.02)	0.28 (0.03)
2a	0.49 (0.02)	0.41 (0.01)	0.24 (0.01)	0.60 (0.03)	0.49 (0.02)	0.28 (0.02)
2b	0.52 (0.02)	0.47 (0.02)	0.31 (0.01)	0.61 (0.02)	0.51 (0.02)	0.31 (0.02)

Standard errors corrected by removing overall between-participant variance are presented in parentheses (Morey, 2008).

**Experiment 1a.** There was a significant effect of perceptual degradation, *F* (1, 23) = 7.46, *p* = 0.01, η_p_^2^ = 0.24, with better memory for blurry (*M* = 0.28) than clear words (*M* = 0.21). There was also a significant effect of boost signal, *F* (1, 23) = 22.23, *p* < 0.001, η_p_^2^ = 0.49, with better memory on target trials (*M* = 0.29) than distractor trials (*M* = 0.20). The interaction between perceptual degradation and boost signal was not significant, *F* (1, 23) = 0.08, *p* = 0.79, η_p_^2^ = 0.003. A Bayesian analysis (Wagenmakers, 2007; Masson, 2011) revealed that the posterior probability of the null hypothesis was 0.82 and the posterior probability of rejecting the null hypothesis was.16, which constitutes positive evidence for the null hypothesis (see also Raftery, 1995).

**Experiment 1b.** There was a significant effect of perceptual degradation, *F* (1, 23) = 16.56, *p* < 0.001, η_p_^2^ = 0.42, with better memory for blurry (*M* = 0.32) than clear words (*M* = 0.22). No effects involving boost signal were significant, as participants were not asked to detect targets in this experiment.

**Comparison of Experiments 1a and 1b.** Attention was divided across tasks (naming and target detection) in Experiment 1a and focused on a single task (naming) in Experiment 1b. To compare results across experiments, corrected hits were submitted to a mixed factor ANOVA that treated experiment (1a/1b) as a between-participants factor. This analysis revealed a main effect of perceptual degradation, *F* (1, 46) = 14.86, *p* < 0.001, η_p_^2^ = 0.24, with better memory for blurry (*M* = 0.30) than clear words (*M* = 0.21). There was also a significant interaction between experiment and boost signal, *F* (1, 46) = 9.70, *p* = 0.003, η_p_^2^ = 0.17. To examine this interaction further, we collapsed across perceptual degradation and compared target and distractor trials across experiments. Corrected hits did not differ across experiments for target trials, *t*(45) = 0.49, *p* = 0.62, d = 0.14 (Experiment 1a: *M* = 0.29; Experiment 1b: *M* = 0.27). However, corrected hits for distractor trials were higher in Experiment 1b (*M* = 0.27) than Experiment 1a (*M* = 0.20), *t* (45) = 2.08, *p* = 0.04, d = 0.60.

### Discussion

Several findings from these experiments are worth noting. First, the perceptual degradation effect of Rosner et al. (2015) was replicated successfully. Recognition was better for blurry than clear words in both Experiments 1a and 1b. Second, an ABE was observed in Experiment 1a, with better recognition for target trials than for distractor trials (Swallow and Jiang, 2010). Third, recognition of target words in Experiment 1a (divided attention) was similar to that for corresponding words in Experiment 1b (full attention), whereas recognition of distractor words in Experiment 1a (divided attention) was worse than for corresponding words in Experiment 1a (full attention). This result replicates prior studies showing that the attentional boost lifts performance up to the level of full attention performance but not beyond (but see Swallow and Jiang, 2014a; Mulligan and Spataro, 2015). In light of these findings, the key new result is that the ABE was similar in magnitude for clear and blurry words. This result suggests that the attentional boost and perceptual degradation do not produce redundant effects on recognition (see also Mulligan et al., 2014).

## Experiments 2a and 2b

Experiments 2a and 2b aimed to establish the replicability of the key result from Experiment 1a. Experiment 2a was a direct replication of Experiment 1a, with targets occurring on 20% of study phase trials and distractors occurring on 80% of study phase trials. Experiment 2b was identical to Experiment 1a with the exception that targets and distractors each occurred on 50% of study phase trials. Previous research has shown that the ABE does not depend on targets being more rare than distractors (Swallow and Jiang, 2012), yet most studies of the attentional boost have included a higher proportion of distractor than target trials (Swallow and Jiang, 2010, 2011, 2012, 2014a,b; Spataro et al., 2013, 2015; Mulligan et al., 2014; Mulligan and Spataro, 2015). As such, we predicted that an attentional boost should occur in both experiments. The key issue in both experiments was again whether the ABE would be smaller for blurry trials than for clear trials.

### Method

#### Participants

Thirty-six^2^ undergraduates (31 female) ranging in age from 17 to 29 years (*M* = 18.36, *SD* = 2.00) participated in Experiment 2a and 24 undergraduates (18 female) ranging in age from 18 to 25 years (*M* = 19.00, *SD* = 1.50) participated in Experiment 2b. A sensitivity analysis like that conducted in Experiments 1a and 1b revealed that we could reliably measure effect sizes larger than *f* = 0.33 for Experiment 2a, and larger than *f* = 0.41 for Experiment 2b.

#### Apparatus, stimuli, and procedure

The apparatus, stimuli, and procedure in Experiments 2a and 2b were the same as in Experiment 1a.

#### Design

The design in Experiment 2a was the same as in Experiment 1a. The design in Experiment 2b was similar to that in Experiment 1a with the exception that there were equal proportions of targets and distractors in the study phase, in contrast to the 0.2/0.8 target/distractor proportions used in Experiments 1a and 2a.

### Results

#### Study phase

Target detection sensitivity was again measured by subtracting the false alarm rate from the hit rate separately for clear and blurry items. For Experiment 2a, sensitivity did not differ for clear (*M* = 0.96) and blurry conditions (*M* = 0.94). For Experiment 2b, sensitivity was slightly higher for clear trials (*M* = 0.91) than blurry trials (*M* = 0.88), *t* (23) = 2.37, *p* = 0.03, d = 0.48.

#### Test phase

**Figure 3:**
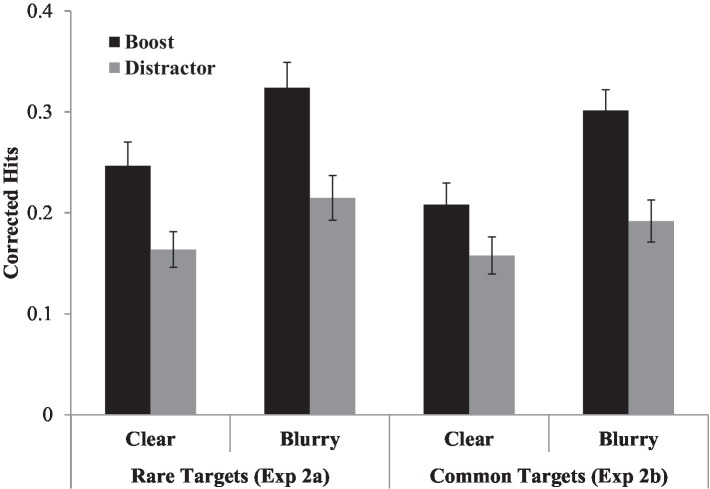
Mean corrected hits (hits minus false alarms) for each condition for both Experiment 2a (20% targets) and Experiment 2b (50% targets). Error bars reflect standard errors corrected to remove overall between-subject variation (Morey, 2008).

For both experiments, corrected hits from the recognition test were submitted to a repeated-measures ANOVA that treated perceptual degradation (clear/blurry) and boost signal (target/distractor) as within-participant factors. Mean corrected hits are presented in Figure 3.

**Experiment 2a**. There was a main effect of perceptual degradation, *F* (1, 35) = 7.83, *p* < 0.01, η_p_^2^ = 0.18, with better memory for blurry words (*M* = 0.27) than clear words (*M* = 0.21). There was also a main effect of boost signal, *F* (1, 35) = 19.59, *p* < 0.001, η_p_^2^ = 0.36, with better memory for target trials (*M* = 0.29) than distractor trials (*M* = 0.19). However, these factors did not interact, *F* (1, 35) = 0.35, *p* = 0.56, η_p_^2^ = 0.01. A Bayesian analysis (Wagenmakers, 2007; Masson, 2011) revealed a posterior probability of the null hypothesis of 0.83 and a posterior probability of the alternative hypothesis of 0.17, indicating positive evidence for the null hypothesis (see also Raftery, 1995). We further explored intraindividual differences in these effects by computing a Pearson correlation coefficient to assess the relation between the perceptual degradation effect and boost effect across all participants in Experiments 1a and 2a. This correlation was not significant, *r* (58) = −0.04, *p* = 0.79.

**Experiment 2b.** There was a significant effect of perceptual degradation, *F* (1, 23) = 5.33, *p* = 0.03, η_p_^2^ = 0.19, with better memory for blurry (*M* = 0.25) than clear words (*M* = 0.18). The effect of boost signal was also significant, *F* (1, 23) = 23.31, *p* < 0.001, η_p_^2^ = 0.50, with better recognition for target trials (*M* = 0.25) than distractor trials (*M* = 0.17). There was also a significant interaction between perceptual degradation and boost signal, *F* (1, 23) = 4.45, *p* = 0.05, η_p_^2^ = 0.16. We explored this interaction further by analyzing boost signal effects separately for clear and blurry items. For clear items, recognition was better for target (*M* = 0.21) than distractor (*M* = 0.16) trials, *t* (23) = 2.43, *p* = 0.02, d = 0.49. For blurry items, the same pattern was observed, with better recognition for target (*M* = 0.30) than distractor (*M* = 0.19) trials, *t* (23) = 4.83, *p* < 0.001, d = 0.90. Critically, the difference in memory sensitivity between targets and distractors was larger for blurry than clear words.

### Discussion

The results of Experiment 2a provide a replication of the results of Experiment 1a. There was an effect of both perceptual degradation and attentional boost, however these factors did not interact. The results from Experiment 2b, in which targets occurred on 50% rather than 20% of trials, differed slightly from those of Experiment 1a. In addition to main effects of perceptual degradation and the boost signal, we also observed an interaction between these two factors. This interaction was driven by a larger ABE for blurry than for clear words. This pattern is not consistent with the idea that perceptual degradation and attentional boost produce redundant effects on recognition memory. If that were the case, then the attentional boost should have been smaller for blurry than clear trials, rather than the reverse.

## General discussion

The purpose of this study was to evaluate the combined influence of target detection and perceptual degradation at study on recognition performance. On their own, both target detection (Swallow and Jiang, 2010) and perceptual degradation (Rosner et al., 2015) at study improve recognition memory. It seemed possible that measuring them in the same experiment would produce redundant effects on recognition, with the ABE being smaller for blurry than clear items. Mulligan et al. (2014) reported an effect of this type for word frequency, with the attentional boost being smaller for low than high frequency words. Which they attributed to redundant attention effects of target detection and word frequency on an early phase of encoding. The present study failed to produce evidence for the predicted interaction. Instead, the ABE was no different for clear and blurry words (Experiments 1a and 2a) or larger for blurry than clear words (Experiment 2b). We conclude that perceptual degradation does not interact with the attentional boost in a manner that suggests redundant attention processes on an early phase of encoding.

Yet, the present results do not rule out the idea that perceptual degradation and target detection affect similar attention processes. Consider that encountering either a boost target signal or a blurry word could draw upon the same limited pool of attentional resources (Wickens, 1980). If resource allocation in response to target detection leaves sufficient resources in the pool that resource allocation in response to a blurry word is unaffected, then the effects of these two variables on recognition would be additive, as observed in Experiments 1a and 2a. Alternatively, the allocation of attentional resources from a limited pool could occur at distinct points in time for target detection and perception of a blurry word, which could also produce additive effects of boost signal and perceptual degradation.

Whether an attentional resource account of this type fits with the word frequency findings reported by Mulligan et al. (2014) is unclear. The interaction between attentional boost and word frequency effects reported in that study would imply either that low frequency words draw sufficient resources from the limited pool to compromise the allocation of resources to target detection, and/or that there is substantial overlap in the time course across which resources are drawn in response to low frequency words and target detection. It is worth noting that Prull (2019) did not find an interaction between attentional boost and word frequency. Instead, the magnitude of the ABE was the same for low and high frequency words. To reconcile these results with those reported by Mulligan et al., Prull speculated that perhaps the low frequency words they used were not orthographically distinct enough from the high frequency words to garner the early allocation of attentional resources in a way that would interfere with boost target detection. An alternative account is that the interaction between target detection and word frequency reflects a form of structural redundancy (Wickens, 1980). By this account, the additive effects of boost signal and perceptual degradation in the present study may occur because there is no structural redundancy between mechanisms required for target detection and the perception of blurry words. Further research on the influence of transient shifts of attention on memory encoding is needed to sort out this issue.

In summary, the present study replicates both the attentional boost and perceptual degradation effects, yet offers no evidence of an interaction that would implicate redundant transient attention mechanisms for these two effects. These results contrast with those reported by Mulligan et al. (2014) in their study of the joint effects of attentional boost and word frequency, and invite additional study of links between transient shifts of attention and long-term memory encoding.

## Data availability statement

The raw data supporting the conclusions of this article will be made available by the authors, without undue reservation.

## Ethics statement

The studies involving human participants were reviewed and approved by McMaster Research Ethics Board. The patients/participants provided their written informed consent to participate in this study.

## Author contributions

ML, TR, JO, and BM: conceptualization, methodology. ML: data curation, formal analysis, project administration, software, visualization, and writing – original draft. BM: funding acquisition and supervision. ML and LL: investigation. ML, TR, JO, LL, and BM: writing – review and editing. All authors contributed to the article and approved the submitted version.

## Funding

Financial support for this study was provided by a Natural Sciences and Engineering Research Council of Canada (NSERC) Discovery Grant awarded to Bruce Milliken (RGPIN-2019-07021) and open access to the published study was supported by funds from Goethe Universitaet awarded to Javier Ortiz-Tudela.

## Conflict of interest

The authors declare that the research was conducted in the absence of any commercial or financial relationships that could be construed as a potential conflict of interest.

## Publisher’s note

All claims expressed in this article are solely those of the authors and do not necessarily represent those of their affiliated organizations, or those of the publisher, the editors and the reviewers. Any product that may be evaluated in this article, or claim that may be made by its manufacturer, is not guaranteed or endorsed by the publisher.

## Footnotes

^1^ gimp.org

^2^Multiples of 20 participants were used in all experiments to properly counterbalance the words lists. Our power analysis indicating at least 20 participants were required to measure a large effect size was met in both experiments.
