# Supplementary material for: The attentional boost effect and perceptual degradation: Assessing the influence of attention on recognition memory
Source: Front Psychol. 2022 Nov 18;13:1024498. doi: 10.3389/fpsyg.2022.1024498 (PMC9716879; doi:10.3389/fpsyg.2022.1024498)
Supplement: Supplementary file 1 [file Table_1.docx]

**Appendix A**

Estimates and analyses of recollection and familiarity based on the independence remember-know procedure (Yonelinas, 2002; Yonelinas & Jacoby, 1995). The table includes estimates for each condition across all four experiments. Standard errors corrected by removing overall between-participant variance are in brackets (Morey, 2008).

|  | *Recollection* | | *Familiarity* | |
| --- | --- | --- | --- | --- |
| *Experiment* | *Target* | *Distractor* | *Target* | *Distractor* |
| 1a  Clear  Blurry | .05 (.021)  .05 (.028) | .09 (.022)  .12 (.019) | .18 (.029)  .30 (.030) | .14 (.023)  .18 (.020) |
| 1b  Clear  Blurry | .09 (.023)  .15 (.032) | .18 (.026)  .25 (.025) | .13 (.031)  .17 (.037) | .12 (.024)  .23 (.021) |
| 2a  Clear  Blurry | .06 (.030)  .20 (.034) | .11 (.020)  .15 (.024) | .17 (.034)  .21 (.029) | .12 (.027)  .12 (.027) |
| 2b  Clear  Blurry | .12 (.015)  .21 (.014) | .08 (.016)  .11 (.018) | .15 (.022)  .20 (.021) | .11 (.020)  .13 (.020) |

To assess the contributions of recollection and familiarity to recognition, for each of the experiments we used the independence remember-know (IRK) procedure (Yonelinas, 2002; Yonelinas & Jacoby, 1995). For each experiment, an estimate of recollection was calculated as the proportion of “remember” (i.e., Type A) responses (R) and an estimate of familiarity was calculated as the proportion of “know” (i.e., Type B) responses (K) given that an item was not recollected (K/1-R). The analysis was conducted on corrected hits (hits minus false alarms) for both recollection and familiarity. These corrected hit scores were analyzed using a repeated-measures ANOVA with perceptual degradation (intact/degraded) and boost signal type (target/distractor) as factors.

*Experiment 1a*

For the recollection scores, the analysis revealed no significant effects. There was no difference in recollection for blurry words (*M* = .09) and clear words (*M* = .07), *F* (1, 23) = .77, *p* = .39, η_p_^2^=.03. There was also no difference in recollection for words with target signals (*M* = .05) and distractor signals (*M* = .10), *F* (1, 23) = 2.48, *p* = .13, η_p_^2^=.10. Finally, the interaction between degradation and boost signal also did not reach significance, *F* (1, 23) = 1.66, *p* = .21, η_p_^2^=.07.

For the familiarity scores, there was a significant difference between blurry words (*M* = .24) and clear words (*M* = .16), *F* (1, 23) = 6.94, *p* = .02, η_p_^2^=.23. There was also a significant difference in familiarity for words with target signals (*M* = .24) and distractor signals (*M* = .16), *F* (1, 23) = 17.97, *p* < .001, η_p_^2^=.44. However, these factors did not interact, *F* (1, 23) = 1.99, *p* = .17, η_p_^2^=.08.

*Experiment 1b*

For the recollection scores, blurry words (*M* = .20) were recollected better than clear words (*M* = .14), *F* (1, 23) = 14.36, *p* < .001, η_p_^2^=.38. There was also a significant difference in recollection for words with target signals (*M* = .12) and distractor signals (*M* = .22), *F* (1, 23) = 5.86, *p* = .02, η_p_^2^=.21. However, these factors did not interact, *F* (1, 23) = .15, *p* = .70, η_p_^2^= .006.

For the familiarity scores, there was a difference between blurry words (*M* = .20) and clear words (*M* = .12), *F* (1, 23) = 6.00, *p* = .02, η_p_^2^=.21. However, there was no such difference between words with target signals (*M* = .15) and distractor signals (*M* = .17), *F* (1, 23) = .70, *p* = .41, η_p_^2^=.03. There was also no interaction between these factors, *F* (1, 23) = 2.13, *p* = .16, η_p_^2^ = .08.

*Experiment 2a*

For Experiment 2a, the analysis of the recollection scores uncovered a difference in performance between blurry words (*M* = .18) and clear words (*M* = .09), *F* (1, 23) = 12.23, *p* = .002, η_p_^2^=.34. However, there was no such difference between words with target signals (*M* = .13) and distractor signals (*M* = .13), *F* (1, 23) = .006, *p* = .94, η_p_^2^< .001. There was a significant interaction between perceptual degradation and boost signal, *F* (1, 23) = 5.46, *p* = .03, η_p_^2^=.19. This interaction was further investigated by analyzing perceptual degradation effects separately for boost target items and boost distractor items. For boost trials, recollection was better for blurry (*M* = .20) than clear words (*M* = .06), *t* (23) = 3.35, *p* = .001, d = .68. The same pattern held for distractor trials, with better recollection for blurry (*M* = .15) than clear (*M* = .11) words, *t* (23) = 2.11, *p* = .02, d = .43.

For the familiarity scores, there was no difference between blurry words (*M* = .17) and clear words (*M* = .14), *F* (1, 23) = .98, *p* = .33, η_p_^2^=.04. However, there was a difference in familiarity scores between words with target signals (*M* = .19) and distractor signals (*M* = .12), *F* (1, 23) = 7.86, *p* = .01, η_p_^2^= .25. These factors did not interact, *F* (1, 23) = .30, *p* = .59, η_p_^2^ = .01.

*Experiment 2b*

For the recollection scores, there was a difference in performance for blurry (*M* = .16) and clear words (*M* = .10), *F* (1, 23) = 10.65, *p* = .003, η_p_^2^= .32. There was also a difference between words with target signals (*M* = .16) and those with distractor signals (*M* = .09), *F* (1, 23) = 17.56, *p* < .001, η_p_^2^= .43. There was also a significant interaction between perceptual degradation and boost signal, *F* (1, 23) = 9.43, *p* = .005, η_p_^2^= .29. This interaction was further investigated by analyzing perceptual degradation effects separately for boost target items and boost distractor items. For boost trials, recollection was better for blurry (*M* = .21) than clear words (*M* = .12), *t* (23) = 5.49, *p* < .001, d = 1.12. However, for distractor trials, there was not difference in recollection between blurry (*M* = .11) and clear (*M* = .08) words, *t* (23) = .83, *p* = .21, d = .17.

For the familiarity scores, there was no difference between blurry (*M* = .16) and clear words (*M* = .13), *F* (1, 23) = 1.65, *p* = .21, η_p_^2^= .07. However, there was a difference in performance for words with target signals (*M* = .17) and distractor signals (*M* = .12), *F* (1, 23) = 11.18, *p* = .003, η_p_^2^= .33. Perceptual degradation and boost signal did not interact, *F* (1, 23) = .51, *p* = .48, η_p_^2^= .02.
