# Supplementary material for: The attentional boost effect and perceptual degradation: Assessing the influence of attention on recognition memory
Source: Front Psychol. 2022 Nov 18;13:1024498. doi: 10.3389/fpsyg.2022.1024498 (PMC9716879; doi:10.3389/fpsyg.2022.1024498)
Supplement: Supplementary file 2 [file Table_2.docx]

**Appendix B**

Word Lists for Experiments 1a, 1b, 2a, and 2b.

*B.1. Word List 1*

TOUCH, RATIO, LEVEL, CHIEF, SWEET, CHEST, GRASS, FIFTY, EMPTY, COUNT, FRONT, BENCH, PEACE, UNDER, SHAPE, LOCAL, OTHER, TREND, ANGER, COVER, FORCE, INDEX, LOOSE, CLASS, VISIT, PLAIN, PROOF, ASIDE, DOING, STYLE, TRADE, OFFER, MUSIC, CAUSE, RIVER, TRIAL, SENSE, EIGHT, STAGE, EARTH, STICK, AWARD, CATCH, PRIME, THIRD, DRESS, ENEMY, PIANO, BIRTH, GUESS, FRAME, PHASE, SKILL, BROWN, MODEL, EXTRA, PLANE, DRIVE, SOUTH, EQUAL

*B.2. Word List 2*

SNAKE, GRACE, NOISE, GRANT, SERVE, UNCLE, NORTH, BOUND, THICK, ROUTE, THING, TEETH, SHEAR, CHILD, POINT, PIECE, UPPER, STAND, STEEL, BRUSH, SHARP, PILOT, SHORT, SMOKE, MAJOR, MATCH, NOVEL, SHORE, TRAIN, PRESS, CURVE, SMILE, STOCK, STONE, IDEAL, CROSS, RADIO, FIELD, BLIND, SPEED, SHARE, PORCH, CROWD, LEAST, DRAMA, AGENT, ORDER, SPOKE, SEVEN, CHAIR, METAL, IMAGE, BAKER, STATE, DROVE, THANK, TOUGH, HORSE, WATCH, BREAK

*B.3. Word List 3*

MONTH, MARCH, WORLD, CLOSE, MAGIC, WHEEL, FOCUS, MORAL, GUEST, QUIET, GROSS, GLASS, PARTY, PRICE, REACH, FIGHT, HURRY, SPACE, APRIL, INNER, KNIFE, STORE, DOUBT, TRUST, GREEN, DEPTH, DRINK, PAPER, GUARD, SOUND, START, BEACH, MONEY, GROUP, CLAIM, WHILE, STORY, SCORE, PRIDE, SPITE, CLEAN, BOARD, TABLE, BLOCK, JUDGE, TRUTH, BRAIN, WAGON, FLOOR, MIGHT, YOUTH, ONSET, STUDY, PHONE, ROUGH, SHEET, TRACK, BRIEF, TITLE, RANGE.

*B.4. Word List 4*

DAILY, SIGHT, ISSUE, RIGHT, QUICK, SCENE, CHAIN, EVENT, HOUSE, STAFF, TODAY, SLEEP, CHOSE, PRIOR, SOLID, GUIDE, WHITE, NIGHT, MOUTH, DREAM, WRONG, THROW, QUEEN, STILL, LEAVE, DOZEN, VOICE, VALUE, SHIFT, MOTOR, SMALL, COAST, CLOTH, LIMIT, PAINT, UNITY, BASIS, WATER, THREE, OPERA, WOMAN, COURT, SMITH, SCALE, THEME, DANCE, PLANT, HEART, TOTAL, ERROR, CHECK, MEANS, MINOR, ROUND, TRUCK, TASTE, BREAD, ANGLE, HOTEL, LIGHT
